# Bogoliubov waves and distant transport of magnon condensate at room temperature

**DOI:** 10.1038/s41467-019-10118-y

**Published:** 2019-06-05

**Authors:** Dmytro A. Bozhko, Alexander J. E. Kreil, Halyna Yu. Musiienko-Shmarova, Alexander A. Serga, Anna Pomyalov, Victor S. L’vov, Burkard Hillebrands

**Affiliations:** 1 Fachbereich Physik and Landesforschungszentrum OPTIMAS, Technische Universität Kaiserslautern, 67663 Kaiserslautern, Germany; 2 Department of Chemical and Biological Physics, Weizmann Institute of Science, Rehovot, 76100 Israel

**Keywords:** Bose-Einstein condensates, Spintronics

## Abstract

A macroscopic collective motion of a Bose–Einstein condensate (BEC) is commonly associated with phenomena such as superconductivity and superfluidity, often generalised by the term supercurrent. Another type of motion of a quantum condensate is second sound—a wave of condensate’s parameters. Recently, we reported on the decay of a BEC of magnons caused by a supercurrent outflow of the BEC from the locally heated area of a room temperature magnetic film. Here, we present the observation of a macroscopic BEC transport mechanism related to the excitation of second sound. The condensed magnons, being propelled out of the heated area, form compact humps of BEC density, which propagate many hundreds of micrometers in the form of distinct second sound—Bogoliubov waves. This discovery advances the physics of quasiparticles and allows for the application of related transport phenomena for low-loss data transfer in magnon spintronics devices.

## Abstract (web summary)

Exploration of magnon Bose–Einstein condensate (BEC) may enable intriguing applications in magnonic devices. Here the authors show experimentally the condensed magnons forming compact humps of BEC density can propagate many hundreds of micrometers in the form of Bogoliubov waves.

## Introduction

Supercurrent is a macroscopic quantum phenomenon, which appears, when many bosons (real- or quasiparticles) being self-assembled in one quantum state with minimum energy and zero velocity—a Bose–Einstein condensate (BEC)^1–9^—move as a whole due to a phase gradient imposed on their joint wave function. This phenomenon, although mostly associated with resistant-free electric currents of Cooper pairs^10^ in superconductors and superfluidity of liquid Helium^11,12^ is, however, much more widespread^13–15^. It is experimentally confirmed in the quantum condensates of diluted ultracold gases^16,17^, of nuclear magnons in liquid ^3^He^18–20^, of polaritons in semiconductor microcavities^21^ and, recently, of electron magnons in room-temperature ferrimagnetic films^22^. Supercurrents being topologically confined often manifest themselves in a form of quantum vortices^16,23,24^.

Another form of motion of the quantum condensates is a second sound^11,25–29^—wave motions of the superfluid parameters, which can propagate in continuous media with an almost linear dispersion law in the long-wavelength limit. From a general viewpoint, this is classified as a specific example of Nambu–Goldstone (NG) modes that appear necessarily in media exhibiting spontaneous breakdown of continuous (for example, translational and/or rotational) symmetries^30^.

The term second sound stems from an analogy with the ordinary sound waves or the first sound—the wave motions of media density and mechanical momentum. The most well-known example is the second sound in the superfluid ^4^He (and in a BEC of cold trapped atoms)—the anti-phase oscillations of the BEC parameters (describing the superfluid component) and of the parameters of excited Bose-particles (describing the normal-fluid component). During second sound propagation, the total density of the supporting media (e.g. superfluid ^4^He) remains constant, *ρ*_He_ = const^11^.

Some solid dielectrics at low temperatures support another type of second sound—the waves of the phonon density^31–34^, with phonons being quanta of the first sound. Here, the phonon density is an analogue of the normal-fluid components in the superfluids. Similarly to the case of ^4^He, during the propagation of this type of second sound, the crystal density *ρ*_cr_ remains constant at large scales.

In the BECs of quasiparticles (e.g. magnons in ferromagnetic dielectrics) one more type of sound may exist—wave motions of the BEC parameters, similar to the fluctuations of the ^4^He superfluid component parameters during the second sound propagation. In the simplest case of the complex order parameter *ψ*(**r**, *t*), these motions are the Bogoliubov waves of the amplitude and the phase of *ψ*(**r**, *t*). Unlike the densities of superfluid and normal components of ^4^He, changes in the density of quasiparticles are unrelated to the density of the supporting media. As a result, during propagation of the magnon Bogoliubov waves, the density of the ferromagnetic crystal remains constant, *ρ*_fm_ = const. Therefore, the Bogoliubov waves in the magnon BEC cannot be associated with the first sound and should be understood as a new type of second sound.

In this paper, by exploring the spatio-temporal dynamics of a magnon BEC prepared by microwave parametric pumping in a room-temperature single-crystal film of a magneto-dielectric material^35–37^, we present the experimental discovery and analytical description of this new type of second sound. Furthermore, we demonstrate a transition from the supercurrent-type to the second-sound-type motion of the magnon BEC. In our experiment, a magnon supercurrent, flowing out from a thermally induced magnetic inhomogeneity^22^, creates perturbations in the BEC density, which travel hundreds of micrometers through the thermally homogeneous film areas almost without changing their form. A detailed analysis of the propagation features of these perturbations in the framework of the Gross-Pitaevskii equation allows us to describe the observed phenomenon as a solitary NG-wave, consisting of the magnon Bogoliubov waves, spontaneously breaking translational symmetry of the magnon BEC. In the long-wavelength limit, realized in our experiment, the Bogoliubov waves have a linear dispersion law and, thus, can be considered as a magnon second sound potentially featuring viscosity-free propagation through the magnon condensate. We consider the discovery of the magnon sound that supports the BEC propagation outside of the area of a strong thermal gradient as the main achievement of this paper.

## Results

### Experiment

The experiment is carried out in a sample (see Methods) cut out from a single-crystal ferrimagnetic film of Yttrium Iron Garnet (YIG)^38^. This magneto-dielectric material possesses the lowest known magnetic damping in nature and is one of the favourite magnetic media for fundamental and applied studies in modern magnonics and spintronics^39^. The detection of magnon dynamics is performed by frequency- and time-resolved Brillouin Light Scattering (BLS) spectroscopy with wavevector sensitivity (see Methods).

Recently, using this method, we succeeded in revealing the presence of a magnon supercurrent by the analysis of an enhanced decay of the magnon BEC in a heated spot created by a probing laser beam^22^. In ref. ^22^ we used a single laser beam both for the heating of the film and for the BLS probing of the magnon BEC. By varying the duration and the power of the probing light we measured and compared time evolutions of the magnon BEC density both in cold and in hot focal laser spots. It was concluded that the only reasonable explanation for the difference between the observed BEC evolutions is based on the assumption of existence of the thermally driven magnon supercurrent flowing from the hot spot to the cold film. This experiment can be considered as indirect observation of the magnon supercurrent, without explicit analysis of its parameters.

**Fig. 1:**
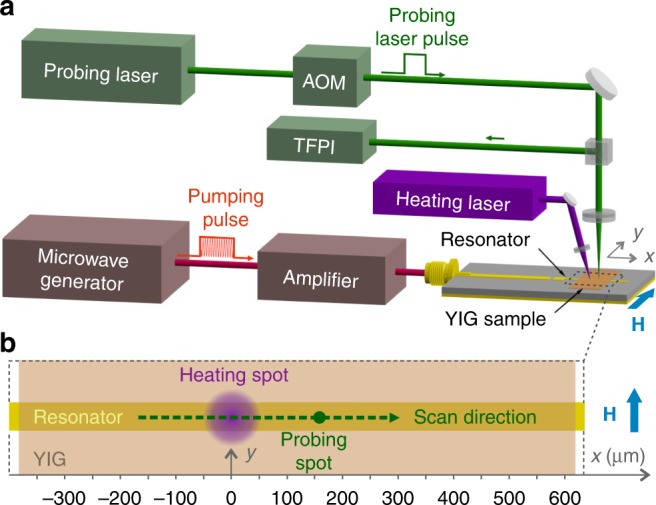
Experimental setup. **a** Schematic illustration of the experimental setup. A probing green laser beam of 532 nm wavelength is focused onto a YIG-film sample, which is fixed on top of a pumping microstrip resonator driven by powerful microwave pulses. The probing beam is chopped by an acousto-optic modulator (AOM) to reduce parasitic heating of the sample. The light and microwave pulses are synchronized allowing for optical observation of the after-pumping evolution of a magnon BEC. The light scattered by magnons is directed to a tandem Fabry–Pérot interferometer (TFPI) for intensity-, frequency-, and time-domain analysis. A blue laser of 405 nm wavelength is used for local heating of the sample and is focused into a 80 μm spot in the middle point of the resonator. The blue laser source and the YIG sample are mounted on a single movable stage to hold unchanged a predefined position of the heated area on YIG-film surface in the process of sample motion. The motion of the stage relative to the focal green spot allows for the probing of the magnon gas density in different points of the sample. **b** The inset schematically shows a magnified view of the scan area. In order to ensure constant pumping conditions and, thus, spatially uniform density distribution of the magnon BEC in the probing direction, the scan is performed in *x*-direction along the microstrip resonator (across the bias magnetic field **H**)

In our current work, we significantly improved the experimental setup, used in ref. ^22^. Now it enables the detection of temporal changes in the spatial distribution of the magnon density caused by the magnon supercurrent. The main idea underlying the direct observation of the motion of the magnon BEC is to separate the area of supercurrent formation from the area in which it was observed. Two independent light sources are used, one for the heating and the other for the BLS probing of the magnon gas as is shown in Fig. 1a. Light from a powerful blue laser, which is completely absorbed by a 5.6-μm-thick YIG film, is used for the film heating. The green light of low power, which is able to penetrate the YIG film with moderate attenuation, is used for the probing. Both the blue laser source and the YIG sample are mounted together on a movable stage to ensure that the predefined position of the heated area on the YIG-film surface remains unchanged. The stage motion relative to the focal green spot allows for the probing of the magnon gas density in different points of the sample.

**Fig. 2:**
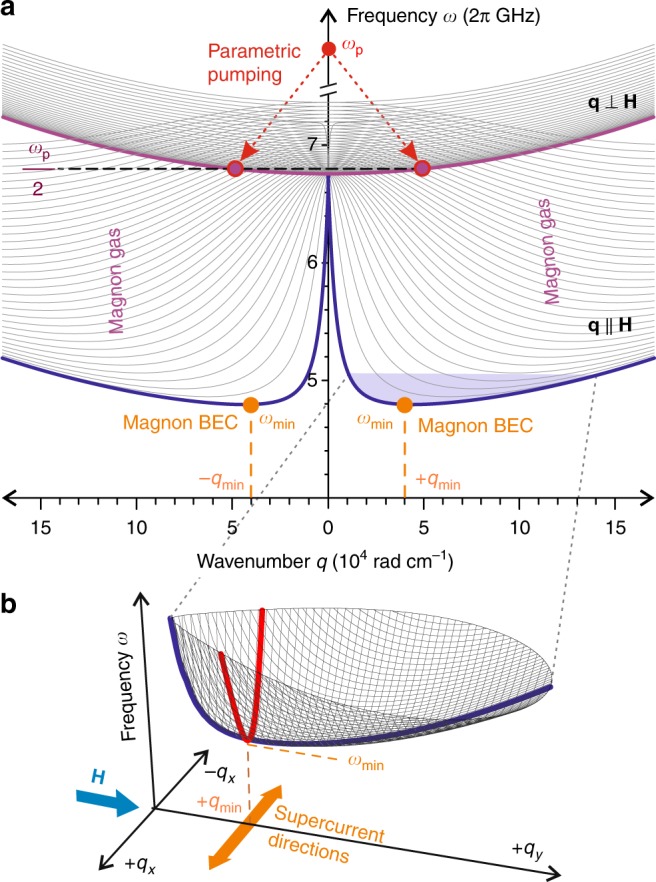
Magnon spectrum. **a** Magnon spectrum of a 5.6-μm-thick YIG film magnetized in plane by a bias magnetic field *H* = 1690 Oe is presented for the wavevectors **q** perpendicular (±**q**⊥**H**) and parallel (±**q**||**H**) to the applied field. For both wavevector orientations the first 35 thickness modes are shown. The red arrows illustrate the process of injection of magnon pairs at *ω*_p_/2 frequency by means of parallel parametric pumping. Thermalization of the parametrically overpopulated magnon gas leads to the formation of Bose–Einstein condensates in two symmetric minima of the frequency spectrum *ω*(**q**) at **q** = ±**q**_min_ (with **q**_min_||**H**). Inset **b** shows the 3-dimensional view of a bottom part of the spectrum (the lowest fundamental mode *n* = 0 within the shaded violet area in **a**) calculated in the range up to 200 MHz above the *ω*_min_. The red curve shows the spectrum of magnons with ±**q**⊥**H** and the blue curve relates to the magnons with ±**q**||**H**. Due to the much higher (about 21 times) steepness of the red curve near (*ω*_min_, **q**_min_), and, thus, proportionally smaller effective masses of the corresponding magnons, the magnon supercurrents predominantly propagate in a film plane across **H** and along the ±**q**_*x*_ directions

In order to achieve BEC^40,41^, magnons are injected into the spin system of the YIG film via parallel parametric pumping^35,37^, which is currently considered the most efficient technique for magnon excitation over a large wavevector range. The process can be described by the splitting of a photon of a pumping electromagnetic wave with a wavevector of nearly zero and a pumping frequency *ω*_p_ into two magnons with opposite wavevectors ±**q** and a frequency of *ω*_p_/2 (see Fig. 2a). The strength of the bias magnetic field *H* of 1690 Oe is chosen to allow for the injection of the magnon pairs slightly above the ferromagnetic resonance frequency *ω*_FMR_, where the parallel pumping process is most efficient^42,43^. The injected quasi-particles thermalize by way of four-magnon scattering processes conserving both their number and the total energy^44–48^. Finally, when the total number of magnons reaches a threshold value, a magnon BEC forms^40,41^. Due to the spatially confined microwave excitation, the BEC is formed in an oblong YIG region located just above the pumping resonator. This quasi-one-dimensional region extends for 50 μm along and for 1 mm across the direction of the bias magnetic field **H** (see Fig. 1b). Due to the large anisotropy of the magnon spectrum (see Fig. 2b), the effective mass of the magnons, which is inversely proportional to the magnon dispersion coefficient *D*(*q*_*x*_, *q*_*y*_) = d^2^*ω*(**q**)/(2d**q**^2^), strongly depends on the orientation of the magnon wavevector **q**. The mass of the magnons with wavevectors placed perpendicular to the bias magnetic field is ~21 times smaller than for magnons having wavevectors along the field^22^. The magnon supercurrent is therefore expected to be ~21 times stronger across the biased magnetic field and thus, along a large extent of the condensate. Taking into consideration these facts, we currently restrict ourselves only to one-dimensional scanning, along the pumping resonator (Fig. 1b).

**Fig. 3:**
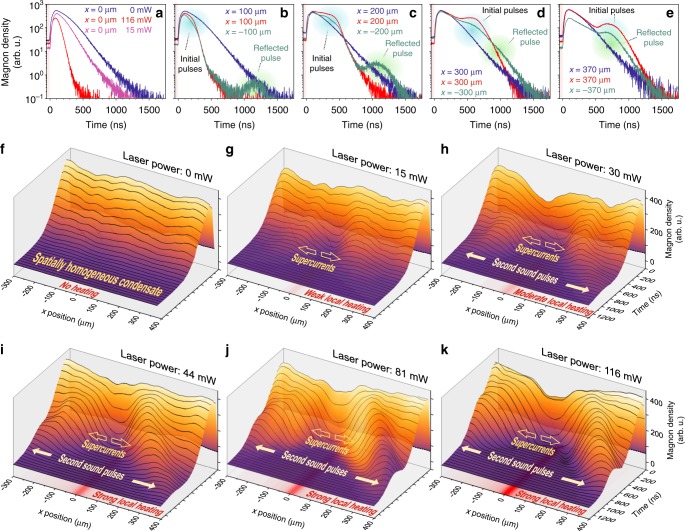
Time and time-space population diagrams of the magnon Bose–Einstein condensate (BEC). **a**–**e** Time evolutions of the temperature-uniform (blue curves) and locally heated (red and green curves for the laser power *P*_L_ = 116 mW, and magenta curve for *P*_L_ = 15 mW) magnon BECs are shown in different spatial positions along the microstrip resonator. The vertical pink stripes mark the end part of the pumping pulse, which is switched off at zero moment of time. Formation of humps of magnon density (marked by smeared blue ovals) and their propagation outwards the hot sample area are clearly visible on the background of a freely decaying BEC. The density hump reflected from the left edge of the sample back to the hot area is visible on the green curves in **b**–**e** (marked by smeared green ovals). In **e** the input and reflected pulses measured just near the left sample edge are overlapped in time. **f**–**k** Space-time evolutions of the magnon BEC after termination of the pumping pulse. After the parametric pumping is switched off at *t* = 0, a BEC density peak is formed. **f** Without external heating: the BEC density exponentially decays in time uniformly over space (cf. the blue curves shown in **a**–**e** in logarithmic scale). **g**–**k** With external continuous heating by a blue laser: the heating does not change the spatial distribution of the magnon density in the course of pumping action (see the dotted curve at the zero moment of time). However, the magnon BEC is pushed out from the hot focal spot (the simulated temperature profiles are shown by red shading around *x* = 0 in the planes below) and a magnon density hole surrounded by two magnon density humps appears in the heated area. At weak heating (**g**), the humps are localized around the heated area. The stronger heating (**h**–**k**) results in the formation of denser humps, which propagate in opposite directions along the microstrip and perpendicularly to the bias magnetic field **H** through the cold magnon BEC over distances of hundred micrometers

The results of the spatially-resolved measurements of the magnon density evolution at the bottom of the spin-wave spectrum are shown in Fig. 3 for different heating conditions. To understand the dynamics of the magnon BEC, we first establish how it behaves in a spatially uniform room-temperature profile. The reference measurement, performed without heating, shows that the spatial distribution of the magnon condensate along the microstrip resonator is uniform (see BEC time profiles shown by the blue curves in Fig. 3a–e and a time-space diagram in Fig. 3f). Similar to the results of ref. ^41^, the BLS signal, which is proportional to the magnon density, rises sharply after the microwave pumping pulse is switched off due to the intensification of the BEC formation process caused by the so-called supercooling mechanism of the magnon gas^41^. Due to the intrinsic magnon relaxation to the phonon bath, the density of the freely-evolving magnon BEC exponentially decreases and we observe a spatially uniform decay of the BLS signal.

We now focus on the temperature-gradient-dependent behaviour of the magnon condensate. The outcome of the experiment is strongly changed when an additional local heating by the blue laser light with power of 15, 30, 44, 81, or 116 mW is applied. The lowest heating power of 15 mW leads to the result expected from the BEC’s decay dynamics^22^: the initially spatially uniform BEC density distribution, created after the termination of the microwave pumping, becomes progressively spatially inhomogeneous due to the enhanced decay of the BEC density in the centre of the heated area (see the magenta curve in Fig. 3a and time-space diagram in Fig. 3g). According to the model proposed in ref. ^22^, this decay is caused by the thermally driven magnon supercurrent flowing to the colder film areas. The experimental data presented in Fig. 3g unambiguously corroborate this model: being pushed out from the centre of the hot spot in the directions indicated by two empty arrows, the BEC phase accumulates around the hot spot. This results in two spatially localized magnon humps. The position of these humps does not change over time. Thus, we can conclude that in this case the magnon supercurrents vanish in the close vicinity of the hot spot.

**Fig. 4:**
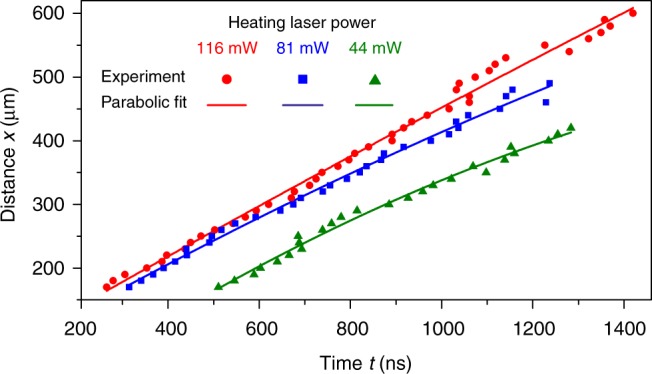
Space-time diagrams of the second sound pulses. Position *x* of the propagating BEC hump is shown vs time *t* for different laser powers *P*_L_ together with parabolic fits, from which we estimate the initial *c*_s_(*t*_0_) and final pulse velocities *c*_s_(*t*_max_) presented in Table 1

At higher heating power, the BEC behaviour changes drastically: after some time elapses, two magnon density humps are identified travelling outwards from the hot spot. The red curves in Fig. 3a–e and time-space diagrams in Fig. 3h–k exemplify the BEC dynamics measured for higher laser powers. Note that the heating is applied continuously, thus unlike in ref. ^22^ the condensate forms in a predefined stable and strong non-uniform temperature profile, which does not change in the process of the BEC formation and the development of the supercurrent magnon flow. As a result, all effects become more pronounced (cf. the relaxation rates of the blue, magenta and red curves in Fig. 3a). In particular, we observe a two and a half orders of magnitude larger decay of the magnon density in the BEC regime (for the maximal heating power of 116 mW, see the red curve in Fig. 3a). To compare, in refs. ^22,48^ the BEC has decayed over only about two decades during the same time interval. Under these heating conditions, the magnon density distribution becomes spatially inhomogeneous already during the condensate formation and the deep magnon density hole surrounded by the dense magnon humps (see Fig. 3h–k) rapidly appear due to the strong magnon outflow. Notably, these humps do not remain at the border of the hot spot but continue to move in the sample areas with rather uniform temperature landscapes. The directions of this motion is marked in Fig. 3h–k by the filled arrows. As is shown by the simulated temperature distributions (see Methods) represented by the red shadings around *x* = 0 in Fig. 3g–k, even the strongest heating by the blue laser is rather local and does not extend beyond ±50 μm from the centre of the focal spot. At the same time, the humps, visible in Fig. 3j, k, propagate to more than 400 μm. The maximum propagation distance observed in our experiment is 600 μm, being restricted by the right edge of the YIG sample. Moreover, one of the density humps is efficiently reflected from the nearby YIG-film edge on the left side of the sample (see Fig. 1b) and propagates hundreds of microns back towards the hot spot (see the corresponding pulses on green curves in Fig. 3b–d). Such a dynamics is common for all heating powers from 30 mW to 116 mW shown in Fig. 3h–k. Stronger laser power leads to a faster formation of more intense BEC humps but does not changes the initial velocities of these humps (see Fig. 4), which is ~400 ms^−1^.

The observed distant propagation of magnon density humps through the uniform BEC cannot be understood using the model assuming the accumulation of a BEC phase in a thermally created magnetic potential and a subsequent supercurrent^22^. Indeed, (i) these humps propagate in the film areas which are subject to rather weak temperature gradients; (ii) the reflected magnon humps move towards the hot spot and thus cannot be driven by the temperature gradient.

### Bogoliubov waves and time evolution of magnon BEC

To discuss the observed time- and space-evolution of the total number of magnons near the lower end of the frequency spectrum *N*_tot_ = *N*_b_ + *N*_c_, which comprises the gaseous bottom magnons *N*_b_ and condensed magnons *N*_c_, we need to clarify the initial conditions for *N*_b_ and *N*_c_ at the moment of time *t* = +0, immediately after the termination of pumping power. The previous wavevector- and time-resolved BLS studies in ref. ^41^ revealed that strong electromagnetic pumping overheats the magnon gas and can thus prevent the condensation process, which develops rapidly after the pumping is switched off^41,44–46^. In addition, the experimentally observed magnon density is not affected by the local heating during pumping (see the dashed line at *t* = +0 in Fig. 3g-k). It indicates that in the entire pumping area no BEC exists under given experimental conditions before the pumping is switched off. Thus, we can assume that for *t* ≤ 0 *N*_tot_ ≈ *N*_b_ and $N_{\mathrm{c}} \ll N_{\mathrm{b}}$ (or equal to zero).

Our understanding of what happens for $t \,\,\gtrsim \,\,0$ in the presence of the hot spot, where the locally reduced saturation magnetization creates a frequency well, is at an infancy state. Essential progress in this direction requires purposeful experimental studies, which will then serve as a basis for a microscopic analytical theory. For the time being, the experiments can be reasonably well interpreted under a conjecture that fast thermalization of *N*_b_ for *t* > 0 creates a high-intensity condensate mainly (or only) in the hot spot. To find possible reasons for such localization we can speculate that the almost-space-homogeneous chemical potential touches the bottom of the frequency spectrum initially at the hot spot. If so, the magnon BECs with ±q_min_ (see Fig. 2a) will try to escape from this area due to their nonlinear repulsion^49^. As is evident in our analysis of the propagation data (see below), the repulsive frequency shift Ω_NL_ is about one order of magnitude larger than the depth of the frequency well *δω*(*T*), induced by the heating (see Methods). Therefore, the magnon BEC can successfully escape the well, creating second sound pulses that propagate away from the hot spot.

These pulses can be formally represented by a Fourier series of monochromatic waves, which propagate with almost the same velocity due to the linearity of the dispersion law (3) in the small wavevector limit. This explains why the observed density humps propagate practically without changing their shape. Moreover, the travelling monochromatic waves should reflect simultaneously from the sample edge, creating a hump of the same form, propagating in the opposite direction. This is exactly what we observe as a reflected pulse on the green curves in Fig. 3e.

We would like to note that the massive transition of the gaseous bottom magnons to an efflux magnon condensate should significantly reduce their concentration *N*_b_ in the centre of the hot spot, and some diffusion of these quasiparticles towards the hot spot to replace the escaping BEC magnons is expected. One can guess that the effective diffusion coefficient is given by the dispersion coefficient $D_x = \partial ^2\omega ({\mathbf{q}})/(2\partial q_x^2)$ at the minimum frequency, which under the conditions of our experiment is about 7.4 cm^2^ s^−1^. If so, the characteristic speed of the diffusive broadening of the magnon density hole of width *L* (about 100 μm or broader, see Fig. 3) will be approximately *D*_*x*_/*L* ≈ 7.4 ms^−1^ or even smaller for the maximum laser power *P* = 116 mW. This is much smaller than the experimentally determined velocity ≈ 400 ms^−1^ of the propagating BEC humps. Therefore, the diffusion of the gaseous bottom magnons is negligible and cannot compensate for the hump run-away motion, leading to the appearance of the hole in the total magnon density *N*_tot_, as is indeed observed in our experiment (see Fig. 3g).

The problem of propagation of the magnon density humps outside of the hot spot can be solved by analysis of the dynamic behaviour of the magnon BEC confined in a long but narrow area along the microstrip resonator (Fig. 1b) using a one-dimensional version of the Gross-Pitaevskii equation (GPE)^50^:

1 $$i\frac{{\partial C}}{{\partial t}} = - D_x\frac{{\partial ^2C}}{{\partial x^2}} + W|C|^2C.$$

Here *C*(*x*, *t*) is the BEC wave function and *W* is the effective amplitude of the four-wave interaction, responsible for the nonlinear frequency shift. The first term in the right-hand side of Eq. (1) is the density of kinetic energy, and the second one is the density of potential energy in mean field approximation. In our case *W* is positive, corresponding to the repulsive interaction in the system of two interacting BECs^49^.

The stationary solution of GPE (1) has the form

2 $$C_0(x,t) = \sqrt {N_{\mathrm{c}}} {\mathrm{exp}}( - i{\mathrm{\Omega }}_{{\mathrm{NL}}}t),\quad {\mathrm{\Omega }}_{{\mathrm{NL}}} = WN_{\mathrm{c}},$$

where |*C*_0_|^2^ = *N*_c_ is the density of magnons in the BEC state. A small perturbation *c*(*x*, *t*) ∝ exp *i*[*kx* − Ω(*k*)*t*] of the stationary solution (2) is an NG-mode. Its dispersion law found by Bogoliubov has the form^51^:

3 $${\mathrm{\Omega }}(k) = k\sqrt {2D_x{\mathrm{\Omega }}_{{\mathrm{NL}}}(1 + D_xk^2/2{\mathrm{\Omega }}_{{\mathrm{NL}}})} ,$$

where Ω and *k* are the angular frequency and the wavenumber of the perturbation, respectively. In the long-wavelength limit $D_xk^2 \ll 2{\mathrm{\Omega }}_{{\mathrm{NL}}}$ this perturbation obeys a linear dispersion law

4 $$\Omega (k) = c_{\mathrm{s}}k{\kern 1pt} ,\quad c_{\mathrm{s}} = \sqrt {2D_x\Omega _{_{{\mathrm{NL}}}}} {\kern 1pt} ,$$

and is understood as a second sound propagating through the BEC with the velocity *c*_s_. At the same time, for large *k*, the BEC contribution may be neglected and the standard quadratic dispersion law applies near the minimum of the spectra: Ω(*k*) = *D*_*x*_*k*^2^.

To check, which of these two limiting cases is closer to our experimental situation, we measured the width Δ of the magnon density humps at half of their height for different heating powers. These measurements enabled us to estimate the characteristic value of the second sound wavenumbers $k \simeq {\mathrm{\pi }}/{\mathrm{\Delta }}$ (the first spatial Fourier harmonic), which give the main contributions to the propagating pulse, see Table 1. Additionally, for each laser power used for heating, we measured the position *x*(*t*) of the hump maximum as a function of the propagation time *τ* = *t* − *t*_0_, where *t*_0_ is some initial moment of time, for which the density hump is already well shaped and such analysis is therefore possible. In order to reveal a possible dependence of the sound velocity on the propagation time we fitted the experimental data by a second order polynomial function *x*(*t*) = *c*_s_*τ* − *δc*_s_*τ*^2^/2*τ*_max_ (see Fig. 4). Here *τ*_max_ is the maximum propagation time, limited by the length of our sample, *c*_s_ and *δc*_s_ are fitting parameters. Using these fits we can estimate the initial velocity of the propagating hump *c*_s_ (at *t* = *t*_0_, *τ* = 0) and its final velocity *c*_s_ − *δc*_s_ at *τ* = *τ*_max_. The results are given in Table 1.

**Table 1:** The parameters of the BEC density humps

*P*_L_, mW	Δ, μm	*k,* rad cm^−1^	*c*_s_(*t*_0_), m s^−1^	*c*_s_(*t*_max_), m s^−1^
116	44	714	410 ± 19	361 ± 19
81	72	436	380 ± 21	292 ± 20
44	88	357	402 ± 26	204 ± 26

*P*_L_ is the heating laser power, Δ is the full width of the humps at half of their height, *k* = π/Δ is the characteristic wavenumber of the second sound that mainly contribute to the hump profile, *c*_s_(*t*_0_) and *c*_s_(*t*_max_) is the initial and final velocities of the propagating humps determined from the parabolic fits in Fig. 4. The velocities uncertainty is estimated using the standard deviations of the fit coefficients.

Our main finding in Table 1 is that the initial velocity *c*_s_(*t*_0_) of the hump only weakly depends on its wavenumber *k*: it varies by about 8% as *k* changes twice. A natural explanation of this fact is based on the assumption that the propagating hump consists of Bogoliubov waves over the background of the magnon BEC with a linear dispersion law (4). If so, the velocity *c*_s_(*t*_0_) should indeed be *k*-independent. To check whether the long-wavelength limit assumption is really valid in our case, we estimate the value of the nonlinear frequency shift Ω_NL_ (2) and the product *D*_*x*_*k*^2^/2 by taking *c*_s_ = 410 m s^−1^ and *k* ≈ 714 cm^−1^ from Table 1 for *P*_*L*_ = 116 mW. The resulting estimates are ${\mathrm{\Omega }}_{{\mathrm{NL}}} \approx 2{\mathrm{\pi }} \cdot 18.1\,{\mathrm{MHz}}$ and *D*_*x*_*k*^2^/2 ≈ 2π ⋅ 0.3 MHz. It is evident, that the long-wavelength limit is well satisfied, supporting the suggested second sound scenario.

The second argument in favor of the suggested scenario for the propagating humps is the time dependence of their velocity. According to Eqs. (2) and (4), the time dependence of the velocity of magnon second sound is $c_{\mathrm{s}}(t) \propto \sqrt {{\mathrm{\Omega }}_{NL}(t)} \propto \sqrt {N_{\mathrm{c}}(t)}$. During hump propagation, the amplitude of the background condensate number *N*_c_ decays and consequently leads to a decrease in the sound velocity, as indeed observed in the experiment (see Table 1 and Fig. 4). Note, that a similar dynamics of a propagating localized pulse of the Bogoliubov wave was observed in a BEC of ultra-cold trapped atoms^26^. Interestingly, the sound velocity decreases much less than expected from the observed decay rate of the cold condensate. This may be explained by peculiarities of the propagation of an intensive hump, which gathers the condensed magnons in front of it and leaves the deep hole behind. Such an accumulation of condensed magnons in the hump may compensate its natural decay to a large extent. We are not yet in the position to discuss this complicated phenomenon in detail. These studies are on our agenda.

Using the estimated value of Ω_NL_, we can also estimate the coherence length *ξ* that determines the characteristic wavenumber k_*_ = 1/*ξ*, at which the linear dispersion law (4) is changed to the quadratic one for *k* > k_*_. This length $\xi = \sqrt {D_x/\Omega _{{\mathrm{NL}}}} \simeq 2.5\,{\rm{\mu}} {\mathrm{m}}$ is much smaller than the minimal hump width ${\mathrm{\Delta }} \simeq 44\,{\rm{\mu}} {\mathrm{m}}$, which gives the maximal second sound wavelength in our experiment (see Table 1). Therefore, the experimental conditions fully correspond to the linear part (4) of the dispersion law (3). This conclusion is consistent with our observations that the second sound pulse propagates as a whole, without dispersive spreading proportional to d^2^Ω(*k*)/d*k*^2^. On the other hand, the coherent length determines the sizes of possible topological singularities in the magnon BEC. For example, the diameter *a*_0_ of vortex cores in the magnon condensate measured under similar experimental conditions in ref. ^24^ is about 1 μm, which is comparable with our estimation. It is also instructive to compare *ξ* and *a*_0_ with the total propagation length about 600 μm. The order-of-magnitude difference means that we are really observing the distant transport of the magnon BEC. Note that in the experiments with a BEC of diluted cold atoms^16^ the ratio of the system size to the core radius is much smaller.

## Discussion

The results presented in this article, addressing the spatially-resolved probing of the dynamics of a magnon BEC, provide direct evidence of supercurrent-related motion of the condensate outwards from the heated spot. The observed occurrence of the magnon BEC propagation outside of the temperature gradient is associated with the excitation of a new type of second sound: Bogoliubov waves in the magnon BEC condensate. The newly discovered magnon second sound differs from the second sound in dielectrics^31,33,34^, in which the phonons can be described in terms of their occupation numbers only, not taking into account their phases. It also differs from the second sound in superfluid ^4^He and in the BEC in diluted atomic systems, where the wave function describes the distribution of real atoms and not of the quasiparticles, as in our case.

The terms supercurrent and second sound reflect different manifestations of the very same phenomenon—the superfluidity, and both result from the solutions of the same GP equation. Note also that being the solution of GPE, the second sound wave package transports the densities of its integrals of motion: energy, momentum, number of magnetic quasiparticles and, associated with them, spin and magnetic moment. In a space-homogeneous coherent state of BEC in an ideal Bose gas, the entropy is equal to zero. In real physical systems the situation is more complicated and the role of entropy requires a detailed study in each particular case^52^, including the BEC of an interacting magnon gas. Our preliminary educated guess is that the entropy transport by the considered second sound (long Bogoliubov waves of magnon BEC) is negligibly small provided its wavelength is much larger than the coherence length. In this respect it differs from other types of second sounds, such as temperature waves in superfluid ^4^He and solids^11,29,31–33^, that do transport significant amount of entropy. It is important to note that according to Landau’s criterion^51^ Ω(*k*) > *c*_s_*k* (see Eqs. 3 and 4), a BEC with repulsive interaction, as realised in our case, is an inviscid superfluid.

Clearly, the magnon second sound requires further detailed experimental and theoretical investigations. For example, one needs to account for the interactions of the bottom gaseous magnons with the BEC magnons, by formulating a model, analogous to the two-fluid model of superfluid helium^11^. In this model, another NG-type magnon second sound—the waves of the bottom gaseous magnons density—can be found^53,54^. Having said that, we should emphasize that from a practical point of view, the three observed phenomena: (i) the transition from the supercurrent-type to the second-sound-type propagation regime, (ii) the excitation of the second-sound-pulses, and (iii) the possibility of a long-distance spin-transport in the magnon BEC, has already paved a way for the application of magnon macroscopic quantum states for low-loss data transfer and information processing in perspective magnon spintronic devices^55–57^.

## Methods

### Sample

The Yttrium Iron Garnet (YIG, Y_3_Fe_5_O_12_)^38^ sample is 5 mm long and 1  mm wide. The single-crystal YIG film of 5.6 μm thickness has been grown in the (111) crystallographic plane on a Gadolinium Gallium Garnet (GGG, Gd_3_Ga_5_O_12_) substrate by liquid-phase epitaxy^58^ at a Department of Crystal Physics and Technology of the Scientific Research Company Carat (Lviv, Ukraine).

### Experimental setup

A sketch of the experimental setup is shown in Fig. 1a. It consists of microwave and optical parts. The microwave part includes a microwave generator, which is used as a source for pumping pulses (pulse duration 2 μs, repetition time 1 ms, carrier frequency 13.6 GHz) followed by a power amplifier, which drives a microstrip resonator circuit with a peak power of 12.6 W. The 50  μm wide half-wavelength microstrip resonator, fabricated on top of an alumina substrate, is used to further increase the amplitude of the pumping microwave magnetic field and its spatial localization. The YIG sample is positioned on top of the resonator, in the area of the maximum microwave magnetic field.

The optical part (Fig. 1a) is used both for the probing of the magnon BEC by means of Brillouin light scattering spectroscopy as well as for the controlled local heating of the YIG sample. Its main parts are the probing green laser (single-mode, 532 nm wavelength), an acousto-optic modulator (AOM), blue laser for heating (multi-mode, 405 nm wavelength), and a tandem Fabry–Pérot interferometer (TFPI)^59–61^.

An excessive heating of the sample by the probing laser can lead to the formation of a magnon supercurrent^22^. In order to minimize the influence of the probing beam on the magnon dynamics, we utilize an acousto-optic modulator, which is used for chopping the probing beam into pulses, in order to reduce a parasitic heating of the sample. The pulsed probing beam (pulse duration 6 μs, peak power 9 mW) is then focused onto the sample surface into a focal spot of 20 μm in diameter. The scattered light is directed to the multipass TFPI for frequency selection with resolution of 100 MHz. A single photon counting avalanche diode detector is placed at the output of the interferometer. The output of the detector is then connected to a counter module synchronized with a sequence of microwave pulses^62^. Every time the detector registers a photon, this event is recorded to a database which collects the number of photons ensuring a time resolution of 250  ps. The frequency of the interferometer transmission is also recorded, thus providing frequency information for each detected photon.

### Frequency- and wavevector-resolved BLS spectroscopy

Brillouin light scattering (BLS) can be understood as the diffraction of the probing light from a moving Bragg grating created by a magnon mode. Some portion of the scattered light, which is proportional to the number of magnons in this mode, is shifted in frequency by an amount equal to the frequency of the mode. In addition, the diffraction from the grating leads to a transfer of momentum during this process. The in-plane component of the wavevector **q**_L_ of the incident light is inverted by a magnon mode, if the magnon wavenumber *q* satisfies the Bragg condition *q*  =  −2*q*_L_ sin (*θ*), where *θ* is the angle of incidence. Simultaneously, the out-plane wavevector of the probing light is inverted due to its reflection from the metal microstrip underlying a semi-transparent YIG sample. By setting the angle *θ* equal to 9.7°, the selection of in-plane magnons with wavenumbers *q*_*y*_ ≈ 4 ⋅ 10^4^ rad cm^−1^ around one of the minima of magnon spectra (Fig. 2) is implemented in our setup^63^. The wavevector resolution was ±2 ⋅ 10^3^ rad cm^−1^ allowing for a rather selective observation of the magnon dynamics around the energy minimum at *q*_*y*_ = q_min_. It enables us to avoid a possible spurious contribution of travelling magnon-phonon hybrid quasiparticles^47^ in our transport measurements.

### Optical heating of the sample

In order to locally heat the sample in a spatial point separated from the probing spot an additional continuous 405 nm wavelength laser is used. The reasoning behind the choice of this laser wavelength is twofold. Firstly, it allows an efficient heating of the thin YIG film, since the absorption of the light in the YIG layer is inversely proportional to the wavelength of the light. Secondly, the chosen wavelength is well separated from the wavelength of the probing laser, and therefore can be rather easily filtered out to exclude any possible influence on the detection system. The heating beam focused into a focal spot of 80 μm in diameter provides a local increase of the sample temperature by 40 K at the maximum laser power of 116 mW. Spatial-resolved probing of the magnon dynamics is performed by the controlled displacement of the sample using a precise linear positioning stage. The entire stage is placed directly between the poles of the electromagnet, ensuring high field uniformity and stability. Since the heating laser is located on the same stage, the position of the heating spot is fixed relative to the sample and to the excitation circuit (and, thus, relative to the created magnon BEC). As a result, the described setup allows for space-resolved measurements of the magnon dynamics across the heated and cold areas of the sample as it is shown in Fig. 1. The spatial resolution (scanning step) is set to 10 μm, which corresponds to half the probing focal spot size.

### Temperature and BEC frequency shift in the hot spot

The temperature profiles of the heated sample were determined by solving a 3D heat-transfer model of the experimental setup using the COMSOL Multiphysics software (https://www.comsol.com/). Hereby, the conventional heat conduction differential equation is solved under consideration of the boundary conditions applied to the model, the material parameters of the used materials and the applied heat source^22^. Heat is deposited exponentially along the film thickness and has a Gaussian distribution in the film plane reflecting the shape of the laser focal spot. The temperature distributions along the long side of the YIG film sample (see Fig. 1b) are shown by red shadings at the bottoms of Fig. 3g–k. The calculated temperature difference Δ*T* between the centre of the laser focal spot and the cold film for the maximal laser power *P*_L_ of 116 mW is about 40 K. The corresponding frequency difference *δω*(*T*) caused by the temperature induced decrease in the saturation magnetization of an YIG film^22,38,64^ is about 2π ⋅ 4.6 MHz. This value is much smaller than the nonlinear frequency shift Ω_NL_ in our experiment.

## Data Availability

The data that support the plots within this paper and other findings of this study are available from the corresponding author upon reasonable request.
